# Protective Role of St. John’s Wort and Its Components Hyperforin and Hypericin against Diabetes through Inhibition of Inflammatory Signaling: Evidence from In Vitro and In Vivo Studies

**DOI:** 10.3390/ijms21218108

**Published:** 2020-10-30

**Authors:** Michela Novelli, Pellegrino Masiello, Pascale Beffy, Marta Menegazzi

**Affiliations:** 1 Department of Translational Research and New Technologies in Medicine and Surgery, School of Medicine, University of Pisa, 56126 Pisa, Italy; 2 Institute of Clinical Physiology, CNR, 56124 Pisa, Italy; p.beffy@gmail.com; 3 Department of Neuroscience, Biomedicine and Movement Sciences, Biochemistry Section, School of Medicine, University of Verona, 37134 Verona, Italy; marta.menegazzi@univr.it

**Keywords:** St. John’s wort, hyperforin, hypericin, cytokines, inflammatory signaling, pancreatic beta cells, diabetes, obesity, metabolic syndrome, insulin resistance

## Abstract

Diabetes mellitus is a very common chronic disease with progressively increasing prevalence. Besides the well-known autoimmune and inflammatory pathogenesis of type 1 diabetes, in many people, metabolic changes and inappropriate lifestyle favor a subtle chronic inflammatory state that contributes to development of insulin resistance and progressive loss of β-cell function and mass, eventually resulting in metabolic syndrome or overt type 2 diabetes. In this paper, we review the anti-inflammatory effects of the extract of *Hypericum perforatum* L. (St. John’s wort, SJW) and its main active ingredients firstly in representative pathological situations on inflammatory basis and then in pancreatic β cells and in obese or diabetic animal models. The simultaneous and long-lasting inhibition of signal transducer and activator of transcription (STAT)-1, nuclear factor kappa-light-chain-enhancer of activated B cells (NF-κB) and mitogen-activated protein kinases (MAPKs)/c-jun N-terminal kinase (JNK) signaling pathways involved in pro-inflammatory cytokine-induced β-cell dysfunction/death and insulin resistance make SJW particularly suitable for both preventive and therapeutic use in metabolic diseases. Hindrance of inflammatory cytokine signaling is likely dependent on the hyperforin content of SJW extract, but recent data reveal that hypericin can also exert relevant protective effects, mediated by activation of the cyclic adenosine monophosphate (cAMP)/protein kinase cAMP-dependent (PKA)/adenosine monophosphate activated protein kinase (AMPK) pathway, against high-fat-diet-induced metabolic abnormalities. Actually, the mechanisms of action of the two main components of SJW appear complementary, strengthening the efficacy of the plant extract. Careful quantitative analysis of SJW components and suitable dosage, with monitoring of possible drug–drug interaction in a context of remarkable tolerability, are easily achievable pre-requisites for forthcoming clinical applications.

## 1. Introduction

Diabetes mellitus affects 463 million people worldwide, and it is one of the fastest growing health challenges of the twenty-first century, with the number of adults living with diabetes having more than tripled over the past 20 years. International Diabetes Federation estimates that there will be 578 million adults with diabetes by 2030, and 700 million by 2045. Diabetes affects the health of individuals, societies and economies; the yearly health care costs for the disease amount to 760 billion USD [https://www.diabetesatlas.org/upload/resources/2019/IDF_Atlas_9th_Edition_2019.pdf]. One of the major risk factors for type 2 diabetes, by far the most frequent form of the disease, typically developing in adult or advanced age, is obesity [1]. Worldwide obesity has nearly tripled since 1975, with over 650 million of adult people affected in 2016, to which we should add almost 2.0 billion subjects who are overweight. If this trend continues, 60% of the world’s population will be overweight or obese by the year 2030 [https://www.who.int/gho/ncd/risk_factors/overweight/en/WHO Obesity and Overweight—World Health Organization 1 April 2020]. Actually, weight loss per se shows beneficial effects on diabetes prevention and treatment [2], considering that a moderate weight loss obtained through lifestyle intervention in an obese population with impaired glucose tolerance could reduce the incidence of diabetes by 58% [3].

In the last decade, inflammation has been increasingly investigated in the context of metabolic disorders. It has been indeed deemed a key player in the development of both type 1 and type 2 diabetes mellitus (T1D and T2D, respectively) and their complications; however, they are triggered by different pathophysiological conditions. In T1D, pro-inflammatory cytokines such as interferon (IFN)-γ, interleukin (IL)-1β, and tumor necrosis factor (TNF)-α are produced by immune and inflammatory cells infiltrating the endocrine pancreas and are considered crucial factors in the autoimmune-mediated pancreatic β-cell death [4]. Likewise, TNF-*α*, IL-1β and IL-6, and other inflammatory mediators (e.g., monocyte chemoattractant protein 1 (MCP1 or CCL2) are credited to play a relevant pathogenic role for the development of T2D [5]. Indeed, these factors are produced and released into the bloodstream by enlarged adipocytes and infiltrating pro-inflammatory M1-macrophages in the course of a systemic low-grade chronic inflammation taking place in the adipose tissue as a consequence of energy imbalance in obesity or metabolic syndrome [6,7,8,9].

Inflammation is a complex physiological response to foreign organisms or tissue injury aimed at eliminating the causative agent and restoring homeostasis [10]. It is recognized as a protective response of the host essential for survival, but it can also cause harm when dysregulated. Adequate control of inflammation is essential for the preservation of tissue integrity. Defective resolution of this defensive process and “misreading” or persistence of inflammatory signals increase the risk of developing not only chronic inflammatory diseases, such as arthritis and autoimmune disorders, but also complex and wide-spread pathologies, including diabetes.

The current therapies against diabetes aim at optimizing metabolic control and reducing risk of macrovascular and microvascular late complications, but none of them targets underlying mechanisms. Actually, targeting inflammation and its signaling pathways may be a clever strategy to prevent, postpone or manage diabetes mellitus and its complications. This goal can be achieved without necessarily replacing the drugs currently used for treatment, but achieving it can, at least, be attempted with complementary intervention aimed at regulating a primary pathogenic mechanism and possibly favoring reduction of drug dosage and prevention of side effects or complications. In this context, much interest in compounds of vegetal origin has been recently raised, in view of future clinical applications. These non-peptidyl compounds are particularly attractive, as they are non-immunogenic, available for chronic oral administration, and usually devoid of major side effects. Polyphenols have been extensively studied for their beneficial effects in metabolic syndrome and diabetes, as plant-based diet regimens rich in phenolic compounds have been shown to improve insulin secretion and insulin resistance [11,12,13].

In this review, we focus our attention on *Hypericum perforatum* L., also known as St. John’s wort (SJW) and its main active component, hyperforin (HPF), that were shown to remarkably protect pancreatic β cells in vitro against the deleterious effects of immune and inflammatory cytokines by inhibiting multiple phosphorylation steps of cytokine signaling. Furthermore, the inhibitory action of SJW extract and HPF has been verified to be surprisingly long-lasting at the cellular level, making them particularly suitable for both preventive and therapeutic use [14,15]. Quite recently, also hypericin (HYP), another typical bioactive compound contained in SJW, has revealed interesting protective effects against the harmful consequences of lipid dysregulation in the context of obesity, fatty liver disease, and type 2 diabetes [16,17].

## 2. Inflammatory Signaling and Diabetes

The deleterious effects of inflammatory cytokines on β cells were discovered in the mid-1980s and have since been extensively reviewed as related to both type 1 and type 2 diabetes [18,19]. These cytokines can induce the expression of various pro-inflammatory and pro-apoptotic genes essentially through the concomitant activation of the two master regulators of the inflammatory response and apoptosis in β cells: the signal transducer and activator of transcription (STAT)-1 activated by IFN-γ, and the nuclear factor kappa-light-chain-enhancer of activated B cells (NF-κB), activated by IL-1β and TNF-α [19,20]. STAT-1 proteins, which are present in an inactive cytosolic monomeric form, when phosphorylated at Tyr701 residue upon IFN-γ stimulation, via its receptor-associated Janus kinases (JAK)-1/2, form dimers and translocate to the nucleus where they bind to specific sequence elements of the regulatory regions of numerous pro-inflammatory and pro-apoptotic genes [18,21]. NF-κB is likewise inactive in the cytoplasm under basal conditions, bound to the inhibitory molecule IκB. When IκB is phosphorylated by IL-1β- and/or TNF-α-activated IκB kinase (IKK) complex, and subsequently degraded in the proteasome, NF-κB can freely translocate to the nucleus, link to specific DNA consensus sequences and induce transcription of numerous target genes, including the inducible nitric oxide synthase (iNOS), cytokines, chemokines, adhesion molecules, and other inflammatory mediators [22,23]. Phosphorylation of the p65 subunit of NF-κB represents a final step essential for its full transcriptional activity. Actually, it has been extensively shown that NF-κB has a major pro-inflammatory and pro-apoptotic role in β cells [22,24,25]. 

Other signaling molecules activated by cytokines in β cells are the mitogen-activated protein kinases (MAPKs), including extracellular signal-related kinase (ERK)-1/2, p38 and c-jun N-terminal kinase (JNK). Although p38 and ERK1/2 may also play a role [26,27], JNK activation, that can also be induced by metabolic signals like free fatty acids and hyperglycemia, appears one of the most important contributors in induction of β-cell apoptosis [28]. Very interestingly, JNK has been recently indicated as a key mediator of insulin resistance in the transition from obesity to T2D, due to its extensive action in different tissues, including adipose tissue, liver tissue, and skeletal muscles, in all of which it is upregulated during obesity [29,30]. JNK activation, already known to be as relevant as that of NF-κB for M-1 polarization of macrophages during development of obesity [31], is further enhanced in the M1-macrophages exposed to high saturated FFA levels, thereby playing a major role in promotion of obesity-induced inflammation and insulin resistance [32]. In muscle cells, JNK-NF-κB signaling is also responsible of palmitate-induced overexpression and activation of the protein tyrosine phosphatase 1B (PTP1B), another major contributor to development of insulin resistance [33].

There is an additional aspect worthy of consideration—that is, the observed decrease in adenosine monophosphate activated protein kinase (AMPK) activity in macrophages from ob/ob mice [34] as well as in the adipose tissue of obese humans [35], both associated with increased M1 activation and adipose tissue inflammation. Conversely, AMPK activation, likely through its stimulatory effect on fatty acid oxidation, is able to inhibit adipose tissue macrophage inflammation, limit JNK activation and improve insulin resistance [34]. These observations are consistent with the demonstration that AMPKα1 deficient mice, fed a high-fat diet (HFD), gain more weight, and show more severe insulin resistance than controls [36]. Thus, the use of safe pharmacological or natural AMPK activators should be taken into account as a plausible intervention, complementary to balanced nutrition and regular physical activity, to prevent onset or aggravation of metabolic diseases. It is worth noting that the beneficial insulin-sensitizing effect of exercise and two commonly employed classes of anti-diabetic drugs (the biguanides such as metformin and the thiazolidinediones), has been at least partially attributed to their ability to activate AMPK [37].

In the inflammatory state, besides the above mentioned cytokine-elicited signaling, several distinct features contribute to excessive production of reactive oxygen species (ROS), in which we can comprehensively include the associated pro-oxidant products often referred as reactive nitrogen species (RNS). It is worthwhile to remind the reader that β cells are particularly susceptible to ROS because of intrinsic large production due to the high glucose oxidation rate required for stimulus-secretion coupling on one side, and low expression of anti-oxidative enzymes such as catalase, superoxide dismutase and glutathione peroxidase on the other side [38,39]. In both T1D and T2D, additional ROS can be generated through the glycation reaction and the hexosamine pathway [40]. Moreover, pro-inflammatory cytokines are involved in ROS generation by triggering the expression of both NADPH oxidase [41,42] and iNOS, the latter catalyzing the production of nitric oxide (NO) from L-arginine [43] and favoring the formation of the peroxynitrite anion. This anion, in turn, oxidizes sulfhydryl groups in proteins, nitrates amino acids like tyrosine, aggravates lipid peroxidation, and causes DNA strand breaks, eventually leading to severe cell damage [44]. NO has been also shown to decrease endoplasmic reticulum (ER) calcium and induce ER stress and the unfolded protein response in β cells [45,46]. The mechanisms by which ER stress contributes to β-cell apoptosis are not completely clear, but induction of the pro-apoptotic proteins C/EBP homologous protein (CHOP), and death protein 5 (DP5) [47,48], as well as downregulation of the anti-apoptotic protein MCL-1, are supposed to be implicated [49]. Finally, the pro-inflammatory cytokines IL-1β and IFN-γ can aggravate ER stress and contribute to apoptosis by impairing the lysosomal activity and blocking autophagic flux [50].

## 3. Anti-Inflammatory Drugs against Diabetes

The major current therapeutic agents to treat T2D, i.e., sulfonylureas, meglitinides, metformin, glucacon-like peptide-1 agonists, insulin-sensitizing glitazones, sodium-glucose linked transporter type 2 inhibitors gliflozins and insulin when required, all improve metabolic control, but display little, if any, anti-inflammatory effects, with the interesting exception of metformin, whose anti-diabetic mechanisms of action still escape full understanding [51]. Besides its recognized ability to activate AMPK [51], metformin has been indeed shown to inhibit NF-κB activation and the downstream factors involved in the inflammatory response [52]. The above-mentioned drugs act by different mechanisms, e.g., by enhancing insulin secretion, increasing insulin sensitivity, suppressing hepatic glucose production, inhibiting glucose reabsorption by the kidney [53]. Unfortunately, none of these anti-diabetic agents used to control hyperglycemia is able to stop or reverse disease progression and each one can cause serious side effects or comorbidities. For example, treatment of T2D patients with insulin, sulfonylureas and meglitinides is associated with weight gain and risk of hypoglycemia; treatment with thiazolidinediones may cause osteoporosis, fluid retention, urinary bladder cancer, hepatotoxicity and increased risk of heart failure [54,55]. Furthermore, metformin, sulfonylureas, and GLP1 agonists tend to lose effectiveness over time [56]. 

A therapeutic approach for T2D that would act primarily on the inflammatory setting has actually been proposed [57]. Several studies showed that drugs that reduce inflammation, such as non-steroidal anti-inflammatory drugs (NSAIDs) are able to improve glucose-mediated insulin release, glucose tolerance, and reduce insulin resistance in diabetic patients [58,59,60]. However, NSAIDs have well-documented side effects, including osteoporosis, impaired wound healing, high risk for gastrointestinal bleeding and ulcerative lesions, stroke [61,62]. Among NSAIDs, salsalate, a pro-drug of salicylate, has attracted attention because it has shown beneficial effects on glycemia and insulin sensitivity, probably acting through both inhibition of NF-κB signaling and AMPK activation [63], the latter likely due to its protonophore and mitochondrial uncoupling activities [64]. Salsalate has the additional advantage that the bleeding risk associated to its use is lower as compared to other NSAIDs [65]. Two multicentre, placebo-controlled studies have confirmed that salsalate decreases systemic inflammation and improves glucose metabolism in patients with T2D, while being well tolerated [66,67]. In any case, there is a strong interest in identifying new anti-inflammatory agents, including those targeting signal transduction, to improve or replace current therapies [68]. Novel anti-inflammatory drugs included IL-1β receptor antagonists, such as canakinumab, initially considered capable of improving both insulin secretion and tissue insulin sensitivity, but later proved to have insufficient clinical efficacy in clinical trials [69]. Diacerein, another inhibitor of IL-1β used so far in osteoarthritis therapy, is currently being tested in Phase III trials for treatment of T2D (NCT02242149) [70]. Finally, anti-TNF-α compounds like etanercept, infliximab, and adalimumab were also shown to reduce insulin resistance in T2D [71]. 

In the treatment of T1D the primary objective has been correction of hyperglycemia and prevention of its complications. Besides insulin therapy, an approach aimed at preventing or slowing down the persistent auto-immune attack against the residual β cells that escaped primary destruction or underwent regeneration, has been warranted for a long time. Despite advances in medical device technology and large availability of insulin analogues, as well as strenuous attempts to obtain in vitro insulin-producing cells to be transferred into diabetic patients, there is still no fully effective way to yield the fine tuning of secretion and function of the natural hormone or replace the lost β cells in T1D [72]. An immuno-suppressive intervention has been considered, based on the fact that pre-diabetic state can be identified early by the detection of specific autoantibodies. However, immunosuppressive drugs often have serious side effects, including high risk of infections [72]. As inflammatory signaling is necessarily involved in β-cell death in T1D, both general and specific anti-inflammatory agents have been tested, mostly at the onset of the disease. So far, two different IL-1β blockers showed no benefit [73], while golimumab, a TNF-α blocker, is currently being tested in clinical trials (NCT03298542) and monoclonal antibodies against IL-12/23 and IL-6 receptor are under preliminary investigation (NCT02117765; NCT02293837). The goal of this innovative approach would be to provide less aggressive environment, thereby favoring β-cell survival and regeneration. 

Altogether, the use of NSAIDs in the treatment of diabetes is limited by the well-known gastrointestinal, renal and cardiovascular side effects, as mentioned. On the other hand, the recently developed molecularly targeted drugs against pro-inflammatory cytokines and chemokines are selective, but expensive to be produced, difficult to be administered and of doubtful efficacy, perhaps just because of their selectivity. For these reasons, both classical and advanced anti-inflammatory agents are not currently used in prevention or treatment of diabetes. In light of these considerations, an approach combining adequate therapeutic achievements and minimal side effects should be adopted by employing more friendly and tolerable anti-inflammatory low molecular weight compounds of vegetal origin. Actually, natural products and derived active ingredients proved to be effective at very low concentrations in preventing or inhibiting a number of crucial steps of inflammatory signaling can represent achievable alternatives for patients with metabolic syndrome or recently diagnosed diabetes, since these compounds are generally safe, non-immunogenic, orally administrable, inexpensive and easily available worldwide.

## 4. SJW and Its Components

*Hypericum perforatum* L. belongs to the plant family of Hypericaceae (Malpighiales order) [74]. Of the currently recognized about 500 species of *Hypericum* genus, *Hypericum perforatum* is the best known [75]. It is a five-petalled yellow-flowered perennial herbal plant, distributed worldwide and most common in Europe, Asia and western United States. It starts flourishing in June, around the period of St. John Baptiste cult (June 24) and for this it is commonly named St. John’s wort (SJW). Flowers have small black dots that, when rubbed between the fingers, produce a red stain due to the constituent HYP. Closely watched against a light background, the tiny elongated leaves of the plant reveal a number of translucent dots corresponding to oily vegetal glands and resembling minute holes that justify the species name *perforatum*. The aerial parts of the plant (mostly flowering tops and apical leaves) are harvested during the flowering season and commonly used, according to the EC and US Pharmacopoeias, either as crude drug (that is, dried plant material) or in standardized extracts [76]. 

SJW has a long history of use in traditional medicine for treating a diverse range of disorders that includes skin inflammation, respiratory impairment, skin wounds, peptic ulcers, bacterial and viral infections [77,78]. In the past years, it has been mostly employed for the treatment of anxiety and mild/moderate depression instead of the conventional antidepressant drugs, as its main component HPF (and perhaps also the other component HYP, according to some studies [79]) share with these drugs the capability of inhibiting the neuronal reuptake of monoamine neurotransmitters [80,81]. Because of its peculiar biological activities and molecular mechanisms that have been better understood and highlighted quite recently, SJW can be potentially useful for a safe treatment of a number of pathologic conditions in which the inflammatory events play a relevant pathogenic role, including obesity and diabetes. 

SJW extract displays heterogenic composition in bioactive compounds. The most typical ones present in *Hypericum* genus and most abundantly in *Hypericum perforatum* [82] are the prenylated acylphloroglucinol HPF and the naphthodianthrone HYP, at concentrations that in common commercial preparations are about 1–5% and 0.1–0.3%, respectively. Other constituents are present in varying amounts in different SJW preparations (Table 1), e.g., flavonoids such as rutin, hyperoside, isoquercitrin, catechins, quercetin, and various quercetin glycosides; biflavones like biapigenin and amentoflavone; phenylpropanes like chlorogenic and neochlorogenic acid [82,83,84,85]. Each of these constituents is usually present in low amounts, with occasional exceptions mainly due to the ample variability of the primary material (“materia prima”). For instance, rutin can reach significant concentrations in *Hypericum perforatum* of Chinese origin, but on the other hand it is absent in other SJW samples [86]. It is also worth noting that the sum of the amounts of the single minor flavonoid components of SJW may often reach levels high enough to provide a biologically significant antioxidant potential [85].

Notably, it must be emphasized that types and amounts of bio-active components, including HPF and HYP, may vary considerably among different types of SJW preparations due to the geographic origin of the plant, the cultivation method, the vegetative stage, the harvesting period, the part of the plant used (i.e., the proportion of flowers and leaves, that may also change from one year to another), the extraction procedure and other variables [83,86,87]. As regards extraction procedures, common solvents are ethanol, methanol, 2-propanol, ethyl-acetate and carbon dioxide, the first being the most suitable for HYP and the latter for HPF recover [88,89]. Not surprisingly, each extraction method gives a different proportion of SJW components due to the different polarity/affinity of the compounds. In most laboratory and commercial preparations, that are often standardized only for the major ingredients HPF and HYP, SJW is usually extracted using a hydro-alcoholic mixture containing at least 50% ethanol. 

HPF and HYP are the characteristic compounds of SJW, being largely more abundant in this herbal species than in other plants, while for instance rutin, hyperoside, and quercetin are mostly present in a large variety of vegetables and fruits. Rutin, hyperoside, and quercetin have been reported to possess antioxidant, antitumoral, and also antidiabetic properties [80,90,91,92,93], but their putative pharmacological use, as well as that of other flavonoids, is much limited by their scarce bioavailability, mainly due to poor intestinal absorption [91,94,95]. Furthermore, flavonoid physiological and pharmacological effects, including anti-inflammatory activity, are observed in vitro only at quite high doses (usually much more than 10 μM), hardly achievable in vivo [96,97]. 

The acylphloroglucinol HPF, abundantly present in the flowering tops of *Hypericum perforatum* plant, is recognized as the main constituent of the SJW extract responsible for the antidepressant [98] and anti-inflammatory activities [99,100]. Usually, in SJW extract, a small amount of the HPF analog adhyperforin, which has a very similar pharmacological profile, is also present [101]. Importantly, HPF pharmacokinetics has been repeatedly investigated, revealing that the compound is available upon oral ingestion of therapeutic doses of SJW hydroalchoolic extract (3 × 300 mg daily) in healthy volunteers, leading to a maximum HPF plasmatic concentration of 0.28 μM (at 3.5 h after a single dose of the extract containing 5% HPF) and a steady-state concentration of 0.18 μM [102]. The bioavailability of HPF as well as that of HYP, and the achievement of adequate concentrations of both compounds in human plasma after oral dosing of SJW were substantially confirmed in a later study [103]. It is interesting to note that for a standard extract of SJW, containing 3–5% HPF and 0.2–0.3% HYP, the plasma HPF levels are approximately one order of magnitude higher than those of HYP [104]. It should be noticed that, differently from the SJW total extract, in which HPF is found in combination with other compounds, including antioxidant flavonoids, purified HPF is quite sensitive to light and oxygen, and its stability is relatively low [105]. Thus, various HPF-salt preparations [102] and stabilized HPF analogs, such as DCHA-HPF [106,107] or aristoforin [108] have been tested and showed to retain HPF biological activity. 

The naphthodianthrone HYP, a naturally occurring chromophore mainly found in SJW, often associated to the closely related and biosynthetic precursor pseudohypericin [109], is a potent photosensitizing agent. Due to its photodynamic and photocytotoxic properties, light-activated HYP has been quite extensively investigated in oncologic therapy and diagnosis [110]. Although less studied, natural HYP also possesses various biological activities without being activated by light [110], including some recently disclosed features that could be helpful for prevention of metabolic diseases (see below).

## 5. Mechanisms of the Anti-Inflammatory Activities of SJW and Its Components

Many studies have investigated the mechanisms responsible for the anti-inflammatory activity of SJW and its components both in vitro and in vivo. 

### 5.1. Inhibition of Cytokine-Induced Activation of STAT, NF-kB and MAPK Signaling

A major mechanism of the anti-inflammatory action of SJW and HPF relies on the potent and simultaneous blockade of the intracellular signaling pathways elicited by the binding of various cytokines to their receptors. According to a number of in vitro studies carried out in our laboratory on pancreatic β cells, SJW and HPF, in a concentration range of a few micromolar, inhibited the aforementioned activations of STAT-1 and STAT-3 (induced by IFN-γ and IL-6, respectively), NF- κB (by IL-1β and TNF-α) and MAPKs (by various stimuli), so that these factors became unable to shift to the nucleus for binding to DNA and driving expression of target genes. The mechanism of action was based on the concomitant inhibition of multiple phosphorylation steps along the JAK/STAT, NF-κB and MAPK pathways [14,111,112], as detailed below.

In other experiments performed in human colon and lung-derived cell lines exposed to a cytokine mixture in vitro, SJW extract likewise suppressed STAT-1 DNA binding activity, JAK2 phosphorylation and iNOS expression [113]. A study by Hammer et al., using microarray to measure changes in the transcriptome of RAW 264.7 mouse macrophages, also suggested that downregulation of genes involved in the JAK-STAT pathway could account for the anti-inflammatory activity of SJW. Besides STAT-1 inhibition, it was of particular interest that HPF induced increased expression of suppressor of cytokine signaling 3 (SOCS3), a negative regulator of JAK/STAT pathway [114]. Similar inhibitory effects of SJW extract and HPF on a number of factors belonging to at least one of aforementioned major pathways or some relevant target genes have been documented in many studies performed in various cell types and experimental situations both in vitro and in vivo, as detailed below.

### 5.2. Inhibition of Phospholipid-Derived Inflammatory Mediators by SJW and HPF

Among the consequences of the SJW/HPF inhibition on STAT and NF-κB transcriptional activity on target genes, there is the strong suppressive effect on the activities of cyclooxygenase (COX) and 5-lipo-oxygenase (5-LO), key enzymes in the production of the inflammatory eicosanoids prostaglandins, tromboxanes and leucotrienes from arachidonic acid, as assessed in vitro [99]. HPF displayed a remarkable efficacy as a 5-LO inhibitor also in vivo, significantly impairing (at a dose of 4 mg/kg b.w. intraperitoneally) leukotriene B4 formation in pleural exudates of carrageenan-treated rats [100]. Strong inhibition of prostaglandin E2 (PGE-2) generation has been confirmed using either SJW extract in an LPS-stimulated RAW 264.7 mouse macrophage model [115], or HPF in an in vitro assay of LPS-stimulated human whole blood cells [116].

### 5.3. Free Radical Scavenging and Antioxidant Activity of SJW and HPF

It is worth noticing that in many studies, the antioxidant properties of SJW and HPF, by either directly ROS scavenging effect or inducing antioxidant enzymes, were associated with their anti-inflammatory action. Indeed, TNF-α, IL-1β and other inflammatory mediators could trigger or enhance pro-inflammatory responses, including NF-κB activation, also by increasing endogenous ROS level that can be regarded in some way as a bona fide second messenger [117,118]. SJW extract, in fact, was reported to display significant free radical scavenging properties both in vitro and in human placental vein [119]. Feisst observed that HPF, with an IC50 of 0.3 μM, inhibited ROS generation and degranulation in human isolated PMN cells challenged by N-formyl-methionyl-leucyl-phenylalanine (fMLP) [84].

### 5.4. Activation of AMPK by SJW and Its Components

Obesity-linked downregulation of AMPK, a master energy-sensing regulator of metabolism, contributes to trigger and maintain an inflammatory state in adipose and other tissues, so that AMPK activity restoration is another important goal to be achieved for effective prevention or treatment of metabolic diseases. A number of AMPK activating agents have been identified, acting by various mechanisms [120]. Interestingly, a very recent study on the protective effects of HYP, one of the bio-active ingredients of SJW, has documented a peculiar HYP-dependent mechanism of activation resulting in increase of AMPK phosphorylation [17]. It is possible that HPF can also activate AMPK, as reported in tumor HL 60 cells [121,122] via a protonophore effect and ATP reduction, but it is unlikely that this mechanism would operate in non-tumor cells. Another recent study [122] reported activation of AMPK in tumor MCF-7 cells by a SJW extract that was not characterized for its composition. However, it cannot be ruled out that AMPK might be indirectly activated by HPF via the upstream kinase CaMKKβ switched on by an increase in cytoplasmic Ca^2+^ favored by HPF-driven activation of the specific transient receptor potential channel (TRPC6).

### 5.5. Anti-Inflammatory Effects of SJW and HPF Mediated by PXR Activation

We must also consider that HPF, one of the main components of SJW extract, is a high-affinity ligand and activator of the xenobiotic pregnane X receptor (PXR), a nuclear receptor known to induce various sets of genes involved in drug metabolism and excretion, including CYP3A4 and other CYP isoenzymes of the P-450 system, and transporters such as P-glycoprotein (P-gp) [123,124]. PXR activation by HPF-containing SJW extract is not only relevant for the accelerated clearance of a number of co-administered drugs [104], but it could also be for the regulation of several inflammatory conditions like those underlying metabolic diseases [125,126] and some acute or chronic bowel disorders [127]. It is indeed recognized that PXR stimulation exerts beneficial anti-inflammatory effects by inhibiting NF-κB transcriptional activity [128]. There is actually a reciprocal inverse relationship between PXR and inflammatory stimuli, so that cytokine-induced NF-κB activation inhibits PXR and its target genes of P-450 system, whereas pharmacologically activated PXR impairs NF-κB-elicited production of pro-inflammatory factors [128,129]. Such mutual hindrance has been attributed to a direct interfering interaction between the transcriptionally active PXR-retinoid X receptor (RXR) heterodimer and the p-65 NF-κB component [130]. Thus, PXR pharmacological activation is considered a potential tool to treat inflammatory bowel disease and even sepsis [124,127], in which PXR agonists, including HPF, ginsenosides [131] and other natural compounds [128], would have the additional advantage of counterbalancing repression of the P450 drug metabolizing system determined by the high circulating cytokine levels [132]. Furthermore, the established relationship between inflammation and metabolic dysfunction raises the realistic possibility of an advantageous utilization of PXR agonists in metabolic disorders [125]. As also obesity, non-alcoholic fatty liver disease (NAFLD) and T2D negatively affect the activity a number of coenzymes of the P-450 system, including CYP3A4 [126,133,134], SJW treatment, primarily aimed at inhibiting the basic inflammatory state, would result in the added benefit of offsetting the repressed activity of P-450 system, hopefully achieving a balanced drug metabolizing function. 

### 5.6. Neuroprotective Effects of SJW and HPF Mediated by Anti-Inflammatory Mechanisms

An intriguing example of beneficial effects of SJW treatment based on a variety of anti-inflammatory and antioxidant mechanisms regards neuroprotection and putative prevention of neurodegenerative disorders, including Alzheimer’s disease (AD). Encouraging experimental data have been obtained so far, even though the way leading to the goal of clinical application is still long and hard. We want to briefly review some of these results, taking into account that a connection is supposed to exist between cognitive impairment or Alzheimer’s disease and metabolic syndrome, based on adiposity and systemic low-grade inflammation underlying both conditions [135,136]. Actually, in PC12 rat pheochromocytoma cell lines, treated with Aβ oligomers or oxygen peroxide to induce cell injury, HPF increased cell viability and reduced both malondialdehyde production and lactate dehydrogenase release, as measured indices of oxidative stress and cell damage, respectively [137]. In the same study, in a colchicine-induced rat model of AD, the reduced hippocampal levels of anti-oxidant enzymes were restored in a dose-dependent manner by the in vivo administration of HPF, which concomitantly prevented impairment in animals’ memory and learning. Interestingly, these beneficial effects of HPF treatment were associated to downregulation of the increased hippocampal NF-κB and IL-1β levels observed in the model [137]. 

Notably, it has been shown that HPF prevents β-amyloid neurotoxicity and spatial memory impairment in rats injected with amyloid-β-peptide (Aβ) in the hippocampus [138], not only by inhibiting Aβ aggregates formation but also by decreasing the amyloid-β deposits in rats injected with amyloid fibrils or in transgenic Alzheimer’s disease mouse models [138,139,140]. The restriction of Aβ assembly by HPF is most likely due to modulation of amyloid precursor protein (APP) processing, by inhibition of COX1 and 5-LO, both involved in β- and γ-secretase-driven APP cleavage [141,142]. Another mechanism by which HPF-containing SJW extract could reduce β-amyloid accumulation relies upon its reported ability to induce the cerebrovascular expression of the P-glycoprotein (P-gp) transporter in a double transgenic Alzheimer’s disease mouse model [143]. Indeed, P-gp is involved in the export of β-amyloid (in particular Aβ1-40 and Aβ1-42) from the brain into the blood [143,144,145]. Induction of P-gp by SJW had been assessed in liver intestinal mucosa and brain [124,146,147] and could be mediated by the concomitant PXR activation, as documented at the blood-brain barrier [147]. 

It is also worth noticing that the expression of brain P-gp is downregulated in human aging and AD and inversely correlates with Aβ deposition and disease progression [148,149]. One possible mechanisms involves NF-κB, which, after activation through the interaction between Aβ1-42 and the receptor for advanced glycation end-products (RAGE), can repress P-gp gene transcription [150]. Another possibility is again related to neuro-inflammation, that is increasingly being recognized as a driver of AD pathogenesis [151,152]. Indeed, Aβ has been shown to increase the expression of the pro-inflammatory cytokines IL-1β and TNF-α [153], which, along with IL-6, were found to reduce the mRNA expression and function of P-gp in the brain endothelial cells of guinea pigs [154]. Therefore, the beneficial action of SJW and HPF in preserving brain P-gp levels and preventing β-amyloid accumulation and consequent neurodegenerative changes could also be related to their well-established inhibitory effect on cytokine signaling and NF-κB activation.

### 5.7. Beneficial Effects of SJW and HPF in Various Experimental Models of Acute and Chronic Inflammation

SJW and HPF exert potent anti-inflammatory effects in several animal model of acute and chronic inflammation by lowering the expression or activity of inflammatory mediators.

Intratracheal bleomycin challenge represents an acute lung injury model in which production of cytokines, chemokines, and growth factors by activated leukocytes, causes fibroblast proliferation and deposition of extracellular matrix in the parenchyma, leading to pulmonary fibrosis. In this model, intraperitoneal HPF treatment reduced the number of polymorphonuclear neutrophils (PMNs) present in the washed out broncho-alveolar mixed cell types and protected mice from collagen deposition and lung fibrosis [155].

An in vivo study in mice demonstrated that SJW extract reduced paracetamol-elicited rise in plasma concentrations of myeloperoxidase and AST/ALT aminotransferases, as well as of cytokines like IL-1β, TNF-α and IFN-γ, meanwhile normalizing glutathione level [156]. The authors concluded that SJW extract is able to protect mice against paracetamol-induced hepatotoxicity by reducing cytokine production, neutrophil recruitment and oxidative stress [156]. In agreement with these results, Paterniti et al. reported that SJW extract very efficiently counteracted edema and other inflammatory changes in a rodent model of periodontitis, a chronic inflammatory disease of periodontium that also affects the surrounding connective tissue [157]. In particular, SJW extract prevented neutrophil infiltration and inflammation, by curtailing IL-1β production and the intracellular adhesion molecule (ICAM)-1 and P-selectin expressions. SJW also impaired p65 NF-κB subunit nuclear translocation, iNOS expression and nitrotyrosine formation, and exerted an anti-apoptotic effect increasing BCL-2 and decreasing BAX expression levels [157]. 

In addition, SJW displayed a pronounced protective role against gastro-enteric inflammatory injury, as observed in rat gastric mucosa damaged by indomethacin administration [158] or in colonic mucosa after induction of experimental inflammatory bowel disease [159]. SJW was also able to protect rat intestinal epithelial architecture caused by irinotecan-elicited inflammation and SJW pretreatment suppressed increased cytokines IL-1β, TNF-α, IL-6 and IFN-γ induced by irinotecan administration in the intestine and liver [160]. 

In our laboratory, we analyzed the effect of SJW on several animal models of acute inflammation. In a model of lung injury elicited by pleural injection of carrageenan, SJW (30 mg/kg orally) attenuated PMNs infiltration, lipid peroxidation, and TNF-α/IL-1β production. Additionally, the degree of staining for ICAM-1 and nitrotyrosine in immunohistochemical tissue analysis was significantly reduced. These anti-inflammatory effects were associated with decreased NF-κB and STAT-3 DNA binding and eventually led to remarkable protection of mice against lung inflammation [161]. We also demonstrated that SJW extract had beneficial effects in mice with zymosan-induced multiple organ dysfunction syndrome, as it resulted in reduction of peritoneal exudation, PMNs migration, myeloperoxidase activity and pulmonary, intestinal and pancreatic injury [162]. The zymosan-elicited rise in iNOS expression, nitrotyrosine production, transaminases, amylases, lipases, bilirubin and creatinine plasma levels were all markedly reduced by SJW treatment [162]. Furthermore, SJW extract reduced the development of cerulean-induced pancreatitis in mice by lowering the expression of nitrosylated proteins and limiting the poly(ADP-ribose) polymers accumulation in the injured pancreas. SJW was also able to inhibit edema, neutrophil infiltration and ICAM-1 protein expression, finally reducing the mortality of mice at five days after cerulean administration [163]. Similar protective effects of SJW were reported after experimental spinal cord injury in mice [164] and rats [165] and in two models of ischemia and reperfusion [166,167]. Recently, Uslusoy, in an experimental sciatic-nerve injury-induced model, showed that SJW prevented the reduction in main antioxidants (glutathione and glutathione peroxidase), and decreased plasma cytokine levels and muscle caspase 3 and 9 expression levels [168].

In conclusion, SJW extract and HPF can attenuate inflammatory response and subsequent tissue injury in several animal models by modulating a number of potentially harmful processes, including ROS production or exaggerated leukocyte myeloperoxidase activity and by hindering signal transduction elicited by inflammatory mediators.

## 6. Effects of SJW Extract, HPF and HYP in β Cells and Isolated Pancreatic Islets

A series of studies performed in our laboratory intended to verify whether SJW extract and its component HPF could interfere with the mechanism of action of cytokines in β cells, and consequently protect them from cytokine-induced functional impairment and cytotoxicity. We believe that the demonstration that these vegetal compounds could target key mechanisms responsible for cytokine-induced β-cell damage would be helpful in the perspective of their utilization as safe natural compounds for the prevention or limitation of β-cell loss in both T1D and T2D. 

We have explored the in vitro effects of SJW extract and HPF on β-cells using a combination of immune and inflammatory cytokines (IFN-γ/IL-1β/TNF-α), corresponding to those supposed to be released within the islets during the autoimmune insulitis process [14,15,111]. We have demonstrated that in the well-differentiated pancreatic β-cell line INS-1E, SJW extract and HPF (0.5–2 µM) prevented cytokine-induced cell dysfunction, iNOS expression and apoptosis by counteracting both STAT-1 and NF-κB activations [111]. In the same experimental conditions, HYP, whose content in standard in SJW extract is about 0.3%, exerted only a partial inhibition of cytokine-induced STAT-1 and NF-κB activations at concentrations at least 10 times higher than HPF. Other minor components of SJW extract (rutin, quercetin, myricetin) were shown to be ineffective up to 25–50 µM [111]. 

**Figure 1:**
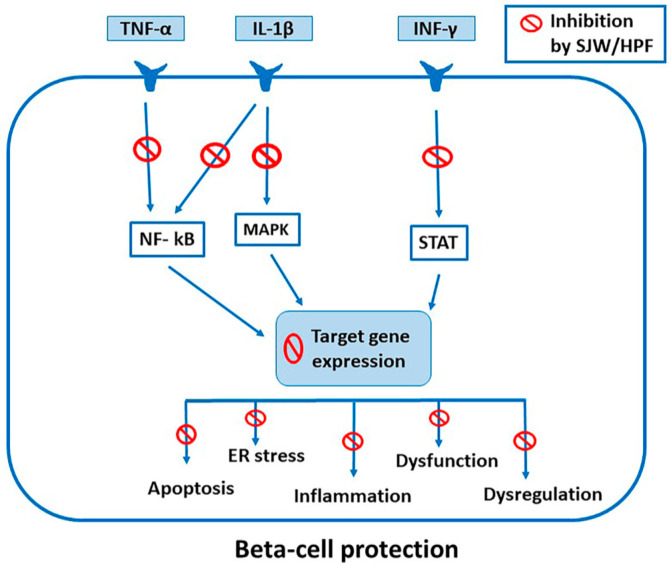
Protective mechanisms of St. John’s wort and hyperforin against cytokine-induced signaling pathways in pancreatic β cells. Abbreviations: TNF-α, tumor necrosis factor α; IL-1β, interleukin-1β; INF-γ, interferon-γ; SJW, St. John’s wort; HPF, hyperforin; NF-κB, nuclear factor kappa-light-chain-enhancer of activated B cells; MAPK, mitogen-activated protein kinase; STAT, signal transducer and activator of transcription; ER, endoplasmic reticulum.

Furthermore, we showed that SJW extract and HPF effectively protect rat and human islets against cytokine-induced β-cell dysfunction, ultrastructural alterations, apoptosis, increased expression of pro-inflammatory genes. We confirmed that the protective effects in the islets were associated with the downregulation of cytokine signaling and transduction pathways, such as STAT-1 and NF-κB [112]. Then, we have shown for the first time that SJW extract and HPF are able to interfere at various levels with the complex and highly regulated pattern of signal transduction and transcriptional activities induced by cytokines in pancreatic β cells. The crucial mechanism of their inhibitory action appeared to be dependent on the simultaneous blockade of multiple phosphorylation steps along STAT-1, NF-κB and MAPK signaling pathways, avoiding or limiting the transcriptional induction of dysfunctional, inflammatory and apoptotic target genes [14] (Figure 1).

With regard to STAT-1 activation, we found that SJW and HPF inhibited both tyrosine-701 and serine-727 STAT-1 phosphorylation. Concerning NF-κB pathway, phosphorylation of both the subunit IκBα activating kinase IKKαβ and the NF-κB p-65 subunit, occurring after 10 and 20 min of exposure to cytokines, were strongly reduced in the presence of the vegetal compounds. Furthermore, a number of upstream kinases involved in IKK activation, including ERK1/2, JNK and Akt, were also inactivated by SJW and HPF [14]. On the other hand, the complete prevention by SJW and HPF of the expression of the pro-inflammatory and immune-enhancer genes tested in INS-1E cells after exposure to cytokines clearly indicates that the vegetal compounds are able to shut out major effectors of the cytokine-driven inflammatory response, such as the iNOS product NO (responsible for harmful oxidative stress and potentiation of pro-apoptotic gene expression), as well as the chemokine (C-X-C motif) ligand (CXCL)-9, CXCL-10 chemokines, ICAM-1 and the class II, major histocompatibility complex, transactivator (CIITA) [14].

In β-cell lines and in isolated rat and human islets, SJW or HPF also prevented cytokine-induced apoptosis, interfering with the expression of genes included in the complex apoptosis-related gene network activated by cytokines [169,170]. The upregulation of the pro-apoptotic factors DP5, p53-regulated mediator of apoptosis (Puma), Bcl2 interacting mediator of cell death (Bim), and Bcl-2 homologous antagonist/killer (Bak) in response to cytokines [47,169,171] was prevented by the vegetal compounds, while the anti-apoptotic factor, B-cell lymphoma-extra-large (Bcl-xL) expression, was increased. The correction of cytokine-driven imbalance between pro- and anti-apoptotic factors was likely dependent on the simultaneous inhibitory effects exerted by SJW and HPF on STAT-1, NF-κB and MAPK pathways. It should also be highlighted that SJW and HPF ward off cytokine-induced ER stress in β cells (as indicated by the full prevention of Chop over-expression), thus neutralizing a supplementary mechanism of inflammatory response and cell death. 

Moreover, it should be highlighted that pre-exposure of cells to HPF induces prolonged changes in the cellular signaling and transcriptional pathways even after HPF is not present any more [15,172]. Indeed, as recently documented in our laboratory, the inhibitory effect of not only SJW extract but also purified HPF on cytokine-induced STAT-1 activation in a pancreatic β-cell line was maintained also after their removal from the incubation medium [15]. In particular, pre-exposure of cells for 2 h to 2 μM HPF was able to markedly inhibit STAT-1 activation even if the compound was washed out before addition of a cytokine mixture (IFN-γ/IL-1β/TNF-α). Furthermore, a similar STAT-1 inhibition was observed when cytokines were added not immediately after HPF removal but 1 h later. As these results confirmed previous observations about the persistence of the effects of SJW extract and HPF in β cells [14,111], we concluded that these compounds undergo an efficient intracellular uptake and confer to the cells a long-lasting state of “cytokine resistance” that does not necessarily require their continuous presence in the extracellular milieu [15]. This property would much improve the bioavailability of SJW components at cellular level and enhance their anti-inflammatory efficacy even at low therapeutic doses.

In a recent study, Liang et al., 2019 [16], dealing with a different condition of in vitro β-cytotoxicity, i.e., that induced by prolonged exposure of INS-1 cell line to high glucose (33 mM for 72 h) and high palmitate (0.2 mM for 24 h) concentrations, reported that HYP, the SJW component only weakly active against inflammatory cytokine signaling, as mentioned above, was actually able to inhibit gluco- and lipo-toxicity-induced apoptosis and NO production. Such protective effects of HYP, exerted at a concentration as low as 0.2 µM, were associated to and probably dependent on a partial restoration of the pancreatic duodenal homeobox (PDX)-1 expression and ERK activity lowered upon high glucose and high palmitate incubation. At our knowledge, HPF, that is able to counteract cytokine induced PDX-1 downregulation, as mentioned above, has not been tested in analogous conditions so far. Thus, it would be very interesting to ascertain whether HPF could afford similar protection against gluco- and lipo-toxicity as HYP or the two main active ingredients of SJW extract exert complementary beneficial effects in jeopardized β cells.

## 7. Effects of SJW Extract and HPF in Adipocytes

Obesity can be considered a state of chronic low-grade inflammation of adipose tissue and is a major risk factor for the development of metabolic syndrome, T2D, non-alcoholic fatty liver disease (NAFLD), hypertension and cardiovascular alterations. It has been observed that during expansion of fat mass a hypoxic state develops in adipose tissue due to a relatively reduced blood flow, resulting in activation of hypoxia-inducible factor-1α and NF-kB with increased expression of pro-inflammatory adipokines [173]. In addition, hypoxia or simply enlarged dysfunctional fat cells can cause adipocyte apoptosis and necrosis [174,175] and consequent elevated secretion of TNF-α and MCP1, that would favor recruitment of macrophages and other immune cells. Therefore, targeting MCP1 emerges as a valid approach to improve metabolism, as confirmed by two recent clinical studies using inhibitors of MCP-1 or its receptor that resulted in amelioration of glycemic control in diabetic patients [176,177].

Actually, as mentioned above, expansion and activation of resident and recruited macrophages within the adipose tissue is considered a major event that occurs during obesity-induced inflammation, resulting in polarization of these macrophage in the M1 pro-inflammatory phenotype [178,179], and consequent increased production and release of TNF-α, IL-1β and IL-6, that largely contribute to the development of local and systemic insulin resistance [8,180,181]. Of note, such cytokines can bind to their receptors on the cell surface of adipocytes and further elevate the expression of pro-inflammatory adipokines, thereby continually driving adipose tissue inflammation [180]. Moreover, cytokines cause JNK activation within adipocytes as well as reduced triglyceride storage and increased lipolysis, leading to elevation of circulating free fatty acids (FFA). In turn, FFA, in particular saturated FFA, can further stimulate JNK and NF-κB pro-inflammatory pathways in adipocytes and macrophages through induction of ER stress or activation of Toll-like receptors on cell surface [182,183]. At the same time, in obesity there is a reduced production of adiponectin, the key insulin-sensitizing adipokine, that regulates glucose and lipid metabolism, activates the energy sensor AMPK and protects against inflammation by inhibiting NF-*κ*B activity and obesity-linked insulin resistance [184,185]. 

Thus, in the chronic inflammatory context mainly due to the dysregulation of adipose tissue in obesity, pancreatic islets are being continuously exposed to increased circulating levels of pro-inflammatory cytokines that can cause functional impairment and progressive loss of β-cell mass, two key features for development of T2D in addition to insulin resistance. Interestingly, it has also been observed that islet cells, including β cells, contribute to initiation and maintenance of islet inflammation, as they can produce and release cytokines, mainly IL-1β, and chemokines in response to stimuli like hyperglycemia, hyperlipidemia, islet amyloid polypeptide (IAPP) accumulation, either directly [186] or through activated intra-islet M1-like macrophages [5].

Despite the known ability of SJW and HPF to inhibit inflammatory signaling in several tissues, a few studies have explored the possibility that SJW extract and its components would indeed inhibit inflammatory response in adipocytes. However, Hatano et al. [187], using adipocyte-derived 3T3-L1 cells showed that SJW extract promoted adipocyte differentiation, increased adiponectin expression via the peroxisome proliferator-activated receptor (PPAR)-γ pathway and prevented TNF-α-mediated expression of MCP-1 and IL-6, through the inhibition of NF-κB activation. 

## 8. Effects of SJW Extract: In Vivo Studies

Many researchers have demonstrated that treatments with SJW extract significantly decrease blood glucose and increase plasma insulin in diabetic animal models. SJW also improves insulin resistance in HFD-fed animals, thus reducing the impact of the diet on obesity or development of diabetes, in the case HFD is prolonged enough (Table 2) [16,188,189,190,191,192,193].

We want to draw attention to the two studies quoted in Table 2 ([16,192]). In [192], Tian et al. treated HFD-induced obese C57BL/6J mice with an extract of *Hypericum perforatum* (EHP) prepared in their laboratory, that mostly contained HYP analogues, as assessed by various analytical methods including HPLC. This extract was able to attenuate glucose and insulin intolerance in HFD-fed mice. Partial improvement of insulin resistance was confirmed by an hyperinsulinemic euglycemic clamp experiment. In skeletal muscles of EHP-treated animals, mRNA and protein levels of protein tyrosine phosphatase 1B (PTP1B), a factor known to be involved in the negative regulation of both insulin and leptin signaling, were found significantly decreased when compared to untreated HFD-fed animals. Furthermore, EHP inhibited the catalytic activity of recombinant human PTP1B in vitro. Serum total cholesterol and triglycerides as well as muscle TG content and expression of the FFA transporter FATP1 were significantly reduced upon EHP treatment as compared to untreated HFD-fed mice. These results suggest that this particular extract, very rich in HYP analogues and apparently devoid of HPF, may improve insulin resistance and lipid metabolism in HFD-fed mice.

Intrigued by the unusual composition (mostly HYP, no detected HPF) of the *Hypericum* extract used by Tian et al. [192], we carefully looked in the literature to find out in vivo studies about the anti-diabetic effects of commercially available purified HPF and HYP, the two main active ingredients of SJW. Whereas no relevant investigation on HPF treatment in animal models of diabetes was found, a recent study, included in Table 1 [16], has yielded interesting results about the in vivo use of HYP in high-fat/high-sucrose (HFHS)-fed mice. In this animal model of metabolic dysfunction, both prophylactic and therapeutic intraperitoneal HYP treatments (in the range 0.5–2 mg/kg b.w.) were able to decrease fasting hyperglycemia and improve glucose and insulin intolerance. Moreover, HYP inhibited β-cell apoptosis and enhanced the antioxidant capabilities of the pancreatic tissue of treated animals, thereby mitigating β-cell loss and preventing or improving overt diabetes. The beneficial effects of in vivo HYP treatment confirmed the observations made in the same study on the in vitro protective action exerted by the compound in β cells exposed for long time to high glucose or palmitate concentrations. 

Even more recently, the same group published the results of a novel study aimed at assessing the protective effects of HYP treatment against the abnormalities in lipid metabolism and the development of NAFLD in HFHS-fed C57BL/6J mice [17] and at elucidating the underlying mechanisms in vivo and in vitro. HYP was administered intraperitoneally every other day at a concentration of 0.5, 1 or 2 mg/kg b.w., for 90 days after one month of HFHS feeding (prevention study) and for 30 days after four months of HFHS feeding (therapeutic study). Preventive use of HYP curtailed the development of NAFLD as well as the abnormalities in lipid metabolism in HFHS diet-fed mice, while its therapeutic use attenuated the severity of these pathological changes. Interestingly, the ex-vivo analysis of the liver removed from both the groups of HYP-treated animals revealed a prevention or reduction of the high oxidative stress parameters caused by HFHS diet and normalization of the lipid metabolism-related proteins, including the phosphorylated form of AMPK (p-AMPK) and the catalytic subunit of protein kinase A (PKACα), found decreased in untreated HFHS-fed animals. The therapeutic use of HYP also alleviated liver malfunction and reduced the occurrence of apoptosis in hepatocytes. These results were paralleled by the in vitro demonstration that HYP could protect hepatocyte cell lines against 1 mM FFA-induced toxicity, by preventing the FFA-dependent intracellular lipid accumulation as well as ROS production and apoptosis. Notably, the improvement of the FFA-induced hepatic lipid alterations occurred via the AMPK pathway, which is one of the most involved in regulation of lipid metabolism [194], that was reactivated by a direct interaction of HYP with the PKACα subunit along the cAMP/PKA upstream regulator pathway, as assessed by molecular docking experiments [17]. Hence, there is evidence that HYP is able to bind PKACα with high affinity and activate it, leading to reversal of FFA-induced lipid metabolism abnormalities in hepatocytes via AMPK signaling. 

Finally, it should be mentioned that SJW administration has been reported to improve glucose tolerance during an oral glucose tolerance test in healthy rats [188,191], but not in young healthy men, in whom impaired tolerance was instead documented, apparently due to reduced insulin secretion in response to the glucose challenge [195]. This latter study has been conducted in a limited number of subjects and needs to be confirmed, but intriguingly the same results have been obtained for other compounds sharing with SJW the ability to activate PXR [196].

## 9. Effects of SJW on Diabetic Complications

SJW has also been shown to prevent diabetic complications, most likely through its anti-inflammatory properties. Actually, as inflammation and oxidative stress play a key role in the initiation and progression of diabetic nephropathy, in streptozotocin-nicotinamide (STZ-NA) diabetic rats, the administration of SJW extract prevented renal functional and structural alterations, while decreasing the kidney expressions of NF-κB protein and inflammatory mediators (TNF-α, IL-1β, ICAM-1, MCP-1), as well as those of the fibrotic marker transforming growth factor (TGF)-β and apoptotic markers [193]. 

Another common complication in diabetes is the impairment in wound healing that can lead to limb amputations, organ dysfunctions, infections, sepsis, and even death. Diabetes affects each phase of the healing process, i.e., haemostasis, inflammatory response, repair and remodeling phase [197]. Various studies have shown that both topical and oral administrations of SJW extract improve tissue healing in diabetic rats [198,199]. Moreover, a topical administration of a mixture of *Hypericum* flower extract and *Azadirachta indica* was able to clearly improve diabetic foot ulcers in a patient with severe diabetes, meanwhile ameliorating metabolic control [200]. The improvement in similar situations has been attributed not only to a reduction in hyperglycemia, but also to an enhancement in fibroblast proliferation, collagen bundle synthesis, and revascularization of skin injuries [201], with the likely contribution of decreased oxidative stress [199]. In this context, it should also be remembered that SJW extract exhibits not only anti-inflammatory but also antimicrobial and antiviral activities [202,203,204,205]. Interestingly, *Hypericum perforatum* oil has been recently incorporated into chitosan films or in bilayer material to create potential new biomaterials that promote wound healing [206,207].

SJW is also capable of modulating pain perception [208]; for this reason, its effects in the painful diabetic peripheral neuropathy, another common complication of this pathology, have been investigated. One-week administration of SJW extract (125 and 250 mg/kg) caused attenuation of mechanical hyperalgesia in STZ-diabetic rats [189]. The efficacy of SJW treatment on prevention of STZ-induced nociceptive behavior was confirmed after acute oral administration of SJW dry extract and it lasted a long time [209]. A number of potential mechanisms can be invoked to explain the anti-nociceptive activity of SJW extract in diabetic animals: anti-hyperglycemic effect, inhibition of nitric oxide synthase activity, impairment in the substance P-induced cytokine synthesis, inhibition of COX and 5-LOX enzyme activities, increased availability of serotonin and noradrenalin levels in supra-spinal descending inhibitory nociceptive pathways, enhancement of γ-aminobutyric acid(GABA)ergic neurotransmission, reduction in the ionic currents of N-methyl-D-aspartate (NMDA) [189]. However, a clinical study aimed at evaluating the efficacy of SJW in diabetic patients suffering from painful polyneuropathy did not confirm the promising results of animal studies [210].

## 10. Discussion

There is large consensus that inflammation is a key pathogenic factor in the development of common and severe pathological conditions such as major metabolic diseases (obesity, non-alcoholic fatty liver disease, metabolic syndrome, type 1 and type 2 diabetes), neurodegenerative disorders, chronic bowel diseases, cardiovascular alterations and several types of tumors. In T1D, the inflammatory process responsible for the progressive destruction of insulin producing pancreatic β cells is well known and stems from an autoimmune reaction selectively targeting such cells within the islets of Langerhans, which are largely infiltrated by activated immune cells either directly cytotoxic or releasing harmful cytokines. Development of T1D is hard to be counteracted because autoimmune process is clinically asymptomatic, even though it can be revealed by the appearance of high-titer specific circulating antibodies. In T2D and in other aforementioned conditions, the inflammatory process runs slowly, at low grade, insidiously, and usually combines with or is triggered by other pathogenic factors often associated with environmental changes and unhealthy lifestyle.

The information available on the effects of SJW in vitro and in animal models has revealed that such extract has diverse preventive and therapeutic potentials and that HPF is in most cases the major bio-active constituent involved in its anti-inflammatory action. However, also HYP has been recently shown to exert beneficial effects in animal models of obesity, NAFLD and T2D, mainly through unexpected modulation of metabolic pathway signaling, that merits mindful consideration, also because it can be complementary to the mechanisms of action of HPF, with the result of a reciprocal reinforcement when both present in the whole SJW extract. With regard to HYP, we refer to the compound naturally present in SJW extract and not to the photoactivated HYP that is employed as antitumor agent and in the treatment of psoriasis.

From the mentioned reports about the protection provided by SJW and HPF against the detrimental effects induced by pro-inflammatory cytokines in various types of cells, included pancreatic β cells, it is clearly apparent that such protection is largely determined by the simultaneous inhibition of STAT-1, NF-κB and MAPK pathways, i.e., the three major signaling and transduction pathways involved in cytokine-induced local and systemic inflammation. Moreover, our recent observation of the persistence of the inhibitory effect of SJW and HPF upon either their removal or their addition following cytokine treatment is helpful for the establishment of their therapeutic potential. Actually, SJW extract and HPF appear to confer to pancreatic β cells a state of “cytokine resistance”, which can be triggered both before and after cytokine exposure and is sufficiently long lasting to rescue β cells from functional impairment and apoptosis.

Among the numerous genes whose expression is markedly downregulated by cytokines in β cells and fully restored by SJW or HPF, besides insulin and glucokinase, there are important homeodomain key factors for maintenance of islet cell differentiated function, such as *Pdx1*, *Nkx2.2*, *Nkx6.1* [14], that can also favor, on a long-term basis, proliferation of islet cells and maintenance or increase in β-cell mass. Cytokine-inhibited Pdx-1 can be also restored by HPF in a different way, i.e., through its known ability to activate TRPC6 channel [211]. 

**Figure 2:**
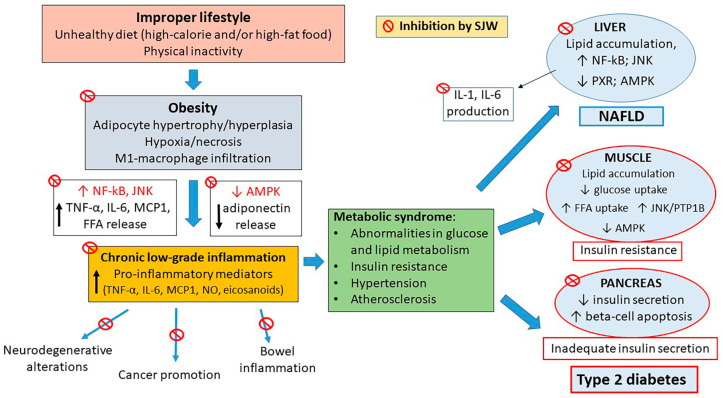
Protective mechanisms of St. John’s wort against development of obesity, metabolic syndrome, non-alcoholic fatty liver disease (NAFLD) and type 2 diabetes due to a chronic low-grade inflammation state. Other diseases sharing, at least partially, an inflammatory pathogenesis are indicated as putative targets of SJW treatment. Abbreviations: SJW, St. John’s wort; M1-macrophages, classically activated M1 macrophages; NF-κB, nuclear factor kappa-light-chain-enhancer of activated B cells; JNK, c-jun N-terminal kinase; PTP1B, protein tyrosine phosphatase 1B; TNF-α, tumor necrosis factor α; IL-1, interleukin-1; IL-6, interleukin-6; MCP-1, monocyte chemoattractant protein-1; FFA, free fatty acids; NO, nitric oxide; AMPK, adenosine monophosphate-activated protein kinase; PXR, pregnane X receptor. ↑: increase; ↓: decrease.

NF-κB plays a crucial role in the inflammatory process underlying development of metabolic diseases, as already highlighted. Actually, in T1D, NF-κB activation in β cells can be induced not only by IL-1β and TNF-α released by infiltrating immune cells during insulitis, but also by β-cell toll-like receptor activation (TLR) and/or ER stress [187]. During development of obesity or T2D, NF-κB activation can be determined by various factors, including chronic release of pro-inflammatory cytokines and chemokines by the enlarged adipose tissue, prolonged exposure to high glucose and advanced glycation end products (AGE), increased free fatty acids levels (via TLR2 and/or TLR4) and/or ER stress [9]. NF-κB signaling and consequent additional production of pro-inflammatory mediators in liver contribute to insulin resistance in the early stages of T2D, whereas activation of NF-κB in adipose tissue M1-macrophages expedites dissemination of circulating inflammatory mediators, promoting systemic insulin resistance in muscle and other insulin-sensitive tissues [9]. Thus, SJW and HPF, through their potent inhibition of NF-κB activation, are expected to block or limit most of the cytokine effects at level of pancreatic islets, liver, adipose tissue and muscle, contributing to prevent/delay the onset of metabolic alterations, if employed earlier, or to reverse/improve them, if given after their occurrence (Figure 2).

Notably, in cytokine-exposed pancreatic β cells, SJW and HPF also inhibit the activation of JNK, a major effector of the MAPK pathway [14], whose role in T2D has been highlighted in a recent review [29]. Actually, JNK can be activated in various tissues by high levels of cytokines and FFA and has been implicated in obesity-induced insulin resistance and insufficient compensatory insulin secretion, two key features of type 2 diabetes, and also associated with diabetic complications. The inhibition of JNK activation by SJW has not been much investigated so far in non-tumor cells and our findings deserve to be confirmed in cell types other than β cells. 

As reminded above, downregulation of the major metabolic regulator AMPK in obesity plays a relevant role in the maintenance of a low-grade inflammation in adipose and other tissues, so that the use of AMPK activating agents has long been considered a promising approach for prevention or treatment of metabolic diseases. A number of AMPK activators have been identified so far [120], many of them acting indirectly by increasing intracellular AMP and ADP (e.g., exercise, salicilate, thiazolidinediones, metformin), others by directly binding to the catalytic α subunit of AMPK (mimetic compounds like 5-aminoimidazole-4-carboxamide-1-beta-d-ribofuranoside AICAR) or to a particular domain of the regulatory subunit β1 close to the kinase domain that is consequently stabilized [212]. Interestingly, also HYP is able to activate AMPK through a peculiar PKA-dependent mechanism, thereby preventing or limiting the impact of HFHS diet on the development of obesity and associated lipid abnormalities, NAFLD and T2D in experimental animals [17] (see Figure 2). It is possible that also HPF can activate AMPK, as reported in tumor HL60 and MCF-7 cells [121,122], via a protonophore effect and ATP reduction, but it is unlikely that this mechanism would operate in non-tumor cells. However, it cannot be ruled out that in normal cells AMPK might be indirectly activated by HPF via the upstream kinase CaMKKβ switched on by an increase in cytoplasmic Ca^2+^ favored by HPF-driven activation of the TRPC6 channel. 

Altogether, the available information allows to summarize that HPF appears to be the most effective component of SJW extract to prevent or counteract the deleterious effects of inflammatory cytokines produced in the islets of Langerhans as a result of an autoimmune attack or released by the enlarged and inflamed adipose tissue in obesity. On the other hand, HYP, the other typical ingredient of SJW, may be also effective for the protection of β cells and other cells involved in the regulation of nutrient metabolism, including adipocytes and hepatocytes, against glucotoxicity and even more against lipotoxicity. In the development of metabolic diseases, altered cytokine production and lipid abnormalities converge to maintain a chronic inflammatory background progressively leading to severe illness. Thus, SJW extract, containing suitable concentrations of both HPF and HYP, might well be an easy, inexpensive and safe alternative or complement to various types of drugs currently in use. The two components of SJW appear actually complementary in the prophylactic or therapeutic use in obesity and T2D. Indeed, HPF would act as a cytokine antagonist and PXR agonist, potently inhibiting inflammatory signaling pathways, while HYP would mainly contribute to prevent or reverse metabolic lipid abnormalities essentially by restoring AMPK activity, depressed by high-fat or high-calorie diet regimens. 

In the perspective of an effective and prolonged use of SJW extract for the prevention or treatment of metabolic diseases, we want actually to stress that caution has to be taken regards the established capacity of SJW (due to HPF content) to activate the liver P-450 drug metabolizing system, through binding of HPF to the PXR receptor. At the dosage required to warrant efficacy against inflammatory signaling, SJW is expected to induce several hepatic CYP isoenzymes, including CYP3A4, primarily active in drug metabolism, possibly leading to accelerated clearance and reduced effect of several co-administered drugs. Thus, upon SJW treatment, careful monitoring of interactions for drugs is recommended, and suitable dosage adjustments of those drugs known to be metabolized through the same enzymatic complexes activated by SJW should be envisaged. However, we should also be aware that not only acute but also chronic inflammatory conditions associated to increased circulating cytokine levels negatively affect the activity of P-450 system [213,214], so that SJW as well as other agonists of the xenobiotic receptor could counterbalance such decline by upregulating CYP expression [127,132]. Actually, it is well known that severe acute and chronic inflammatory diseases such as sepsis, autoimmune disorders like rheumatoid arthritis, and the multi-symptomatic form of the current pandemic coronavirus disease 2019 (COVID-19) markedly depress drug metabolizing system [132,213,215].

Moreover, there are experimental and clinical evidences that also metabolic diseases are associated with an impairment of the P-450 system [134,216]. Indeed, in obesity, CYP3A4-mediated drug elimination was found to be consistently lower among obese as compared with non-obese subjects [126]. In a recent study in diabetic patients, the activities of major CYP coenzymes, including CYP3A4, were decreased by 40–45% [134]. CYP3A4 activity was likewise reduced in human NAFLD as well as in a mouse model of such disease and in vitro cellular models [217]. Gene expression of a number of enzymatic complexes of the P-450 system was found reduced by 30–60% in HFD-fed compared to control mice, associated with downregulation of nuclear receptors such as PXR [218]. Thus, SJW treatment in metabolic diseases, primarily aimed at inhibiting the basic inflammatory state, would have the additional advantage of counterbalancing the aforementioned repression of the P-450 drug metabolizing system and concomitantly reducing the overall risk of abnormal drug interactions. Actually, similar considerations prompted us to propose that SJW might protect COVID-19 patients against the cytokine storm syndrome, simultaneously exerting a compensatory upregulation of the impaired P-450 system [219].

In the course of preparing this paper, we have reviewed plenty of in vitro and in vivo reports dealing with the anti-inflammatory effects of SJW and its main bioactive ingredients, focusing our attention on relevant studies aimed at evaluating the protective action of SJW and its components against development of metabolic diseases. During this work, we have made some general considerations that we would like to share with readers. Firstly, we realized that in a number of in vitro studies the concentrations of plant extracts or bioactive compounds used were very high, quite implausible with respect to those reached following therapeutic administration. We were also negatively surprised that a composite extract like that of SJW was not always titrated with regard at least to its main components, taking also into account the large variability of its qualitative and quantitative content, as pointed out above and shown in Table 1. These observations are in line with the general worries that recently prompted the Editors of the major journals in the phytotherapy field to publish accurate guidelines for best practice in phytopharmacological research [220]. Another consideration regards the fact that we did not find so many experimental in vivo studies exploring the potential of SJW and its components for the prevention or cure of metabolic diseases, probably due to the fact that the anti-inflammatory properties of SJW have been for long time neglected, and also to the underestimation of the pathogenic role of low-grade inflammation in the development of such diseases. Good examples of well-designed in vivo investigations are the two studies of Liang et al. [16,17], discussed above, dealing with the prophylactic and therapeutic use of HYP in a diet-induced model of diabetes and fatty liver disease.

Furthermore, as SJW is one of the most widely used herbal remedies around the world [221], mainly as mood regulator and antidepressant, due to its easy availability, safety and cheapness, we wonder why it has been scarcely studied in humans for its anti-inflammatory potential in metabolic disorders. Actually, SJW has been extensively tested in clinical trials for the treatment of mild-to-moderate depression, resulting as effective as other conventional antidepressants while having less side effects and lower risk of therapy discontinuation [222]. Due to the large number of people with depression taking for long time a daily dosage of SJW extract under medical supervision, it would be of great interest to perform retrospective studies in this patient population to find out whether they could be protected against development or aggravation of obesity, metabolic syndrome and T2D as compared with appropriately matched untreated control subjects. The occurrence and severity of other inflammation-related diseases such as Alzheimer’s disease, rheumatoid arthritis, tumors, inflammatory bowel disorders might be similarly analyzed in SJW-treated patients. A large study of this kind has been actually carried out in USA, with over 77,000 participants followed for five years. This study examined whether the continuative use of various herbal supplements was associated with risk for lung or colorectal cancers. The results showed that regular use of SJW was associated with a 65% decrease in risk for colorectal cancer [223].

## 11. Concluding Remarks and Perspectives

As discussed in this review, inflammation is the common pathogenic mechanism underlying development of T1D, the outcome of an autoimmune reaction against pancreatic β cells, and other metabolic diseases, such as obesity, metabolic syndrome, NAFLD and T2D, resulting from a subtle, low-grade, multi-organ inflammatory process triggered by metabolic abnormalities. While we do not know what triggers the autoimmune reaction leading to T1D, we are well aware of the responsibility of unhealthy lifestyle, above all the excess and/or imbalance in food intake and physical inactivity, in determining obesity and the underlying inflammatory state. In this context, multiple pathways are activated by cytokines and other inflammatory mediators in different tissues and are difficult to be simultaneously targeted by classical pharmaceuticals that usually also engender serious adverse effects. It is well known that plant-derived natural products rich in bioactive compounds of scientifically proved efficacy, such as SJW extract presented here for its powerful anti-inflammatory action, offer a valuable alternative or complement to the classical pharmacological intervention, as they are safer and allow long term treatment, thereby being particularly suitable for a preventive approach. SJW has a number of advantageous features that are not so common among other vegetal compounds with theoretically promising properties, that is an excellent bioavailability and well-established pharmacokinetic parameters, which are crucial in view of clinical applications. Quite peculiar is also the already established therapeutic use of SJW extract as antidepressant, at a dosage corresponding to that required to reach circulating HPF concentrations within the range of those proved to inhibit inflammatory signaling in vitro. The safety profile of SJW is considered remarkable, even though its established ability to induce drug metabolizing system through PXR activation requires careful medical monitoring of drug interactions. However, as mentioned, the inflammatory background of metabolic diseases exerts a repressive effect on drug metabolizing function, that can be advantageously counterbalanced by SJW induction. 

It has actually to be admitted that the interference exerted by SJW and its component HPF on drug metabolism is probably a major reason why SJW has so far been little used in clinical studies, with the relevant exception of depressed patients. Another reason might be the still limited awareness in the scientific community of the remarkable anti-inflammatory potential of SJW. In any case the insufficient clinical investigation carried out to date constitutes a momentary limitation for the exploitation of SJW features, which should soon be remedied. An additional interesting perspective is represented by the finding [224] that a number of HPF analogs are unable to induce PXR and drug metabolism, while maintaining the capability of activating the TRPC6 channel, which is likely involved in the mechanism of HPF anti-depressant activity [225]. These analogs need to be further investigated, to assess if they also conserve the HPF anti-inflammatory and anti-diabetic effects.

Altogether, the properties of SJW discussed in this review prompt to strongly encourage the employment of this plant extract as nutraceutical supplement for both prevention and therapy of metabolic diseases, especially in people at high risk because of genetic background, improper lifestyle, and overweight status. It is certainly recommendable that SJW protection against metabolic diseases would be rigorously evaluated in the near future in controlled clinical trials that can definitely ascertain its prophylactic and therapeutic potential.

## Figures and Tables

**Table 1:** Indicative amounts of most significant constituents of *Hypericum perforatum* phytocomplex detected in different studies, and their predominant localization in the plant.

	Napoli E 2018 [82]	Bruni R 2009 [83]	Seyis F 2020 [84]	
	mg/g Dry Weight	mg/g Dry Weight	mg/g Dry Matter	High Yielding Plant Part
**Naphtodianthrones**				
Pseudohypericin	5.14	0.1–12	0.05–6.75	Dark glands in leaf and petal margin; stamens
Hypericin	3.69	0.1–7	0.01–2.77	
**Acylphloroglucinols**				
Hyperforin	41.0	0.3–150	2.15–28.1	Flowering tops; sepals; translucent glands in leaves
Adhyperforin	4.68			
**Flavonoids**				
Catechins	0.02		1.41–8.7	Floral dehiscent leaves: sepals, stamens, petals. Likely accumulation in vacuoles
Quercetin-3-*O*-galactoside	4.34			
Quercetin-3-*O*-glucoside	1.87			
Quercetin-3-*O*-rhamnoside	2.13			
Quercetin	0.30		0.05–6.04	
Isoquercitrin			0.15–6.99	
Hyperoside		1–25	1.70–22.3	
Rutin		0–35	0	
**Phenylpropanes**				
Chlorogenic acid			0.42–10.55	Flowers and leaves
Neochlorogenic acid			0.37–4.25	
**Biflavones**				
Biapigenin	4.56	0.3–10.2	Trace-2.65	Floral dehiscent leaves: sepals, stamens, petals.
Amentoflavone	0.18	0–1.8		

**Table 2:** Effects of SJW extract in animal models of diabetes.

Dosages	Models	Effects	Hypothesized Mechanisms	Refs.
SJW standard extract 100, 200 and 300 mg/kg b.w. daily oral administration for 14 days	STZ-NA diabetic rats	Dose-dependent reduction of fasting blood glucose levels	Antioxidant and free radical scavenging properties; stimulation by of muscarinic M3 receptor in β cells and increased insulin release; activation by HPF of TRPC6 cation channels and increased glucose-stimulated insulin secretion.	Husain GM 2009 [188]
SJW standard extract 125 or 250 mg/kg b.w. daily i.p. administration for one week	STZ diabetic rats	Dose-dependent decrease in hyperglycemia; restoration of metabolic parameters and improvement of decreased body weights		Can ÖD 2011 [189]
SJW oral suspension in 0.3% carboxy-methyl cellulose 100 and 200 mg/kg b.w. daily for 15 days	High-fat-diet-fed rats Fructose-fed rats	Decrease in plasma glucose and insulin levels; improvement of lipid abnormalities; prevention of weight increase	Reduction of appetite and food intake mediated by serotonin increase.	Husain GM 2011 [190]
SJW ethyl acetate extract 50, 100 and 200 mg/kg b.w. daily i.p. administration for 15 days	STZ diabetic rats	Decrease in blood glucose, serum triglycerides and total cholesterol; increase in plasma insulin and muscle and liver glycogen content	Increase of insulin secretion by the remaining β cells; enhanced muscle and liver glycogen content; decline in glucose-6-phosphatase activity and gluconeogenesis.	Arokiyaraj S 2011 [191]
SJW extract containing mainly hypericin analogues 50 and 200 mg/kg b.w. daily by gastric gavage for three weeks	High-fat-diet-fed C57BL/6J mice	Improvement of hyperinsulinemia, hyperglycemia, insulin tolerance and dyslipidemia	Increase in insulin sensitivity and fatty acid oxidation through PTP1B inhibition.	Tian J 2015 [192]
SJW standard extract 50, 100 and 200 mg/kg b.w. daily by gastric gavage for eight weeks	STZ-NA diabetic rats	Decrease in hyperglycemia and increase in insulinemia; protection against nephropathy	Same mechanisms as in [191] and [192].	Abd El Motteleb 2017 [193]
Hypericin 0.5–2 mg/kg b.w. daily i.p. administration for either 90 or 30 days	High-fat/high-sucrose-fed mice	Prevention in weight gain; decrease in fasting hyperglycemia; improvement of glucose and insulin intolerance.	Reduction of gluco- and lipo-toxicity; improvement in β-cell function; maintenance of β-cell mass; prevention of insulin resistance	Liang C 2019 [16]

Abbreviations: SJW, St. John’s wort; HPF, hyperforin; STZ, streptozotocin; STZ-NA streptozotocin-nicotinamide; TRPC6, transient receptor potential channel; PTP1B, protein tyrosine phosphatase 1B; i.p, intraperitoneal; b.w., body weight.

## Abbreviations

T1D	type 1 diabetes mellitus
T2D	type 2 diabetes mellitus
IFN	interferon
IL	interleukin
TNF	tumor necrosis factor
MCP-1	monocyte chemoattractant protein-1
M1-macrophages	classically activated M1 macrophages
SJW	St. John’s wort
HPF	hyperforin
HYP	hypericin
STAT	signal transducer and activator of transcription
NF-κB	nuclear factor kappa-light-chain-enhancer of activated B cells
JAK	receptor-associated Janus kinases
IkB	inhibitor molecules
IKK	protein kinase complex
iNOS	inducible nitric oxide synthase
MAPKs	mitogen-activated protein kinases
ERK	extracellular signal-related kinase
JNK	c-jun *N*-terminal kinase
ROS	reactive oxygen species
RNS	reactive nitrogen species
ER	endoplasmic reticulum
CHOP	C/EBP homologous protein
DP5	death protein 5
NSAIDs	non-steroidal anti-inflammatory drugs
AMPK	adenosine monophosphate-activated protein kinase
SOCS3	suppressor of cytokine signaling 3
DCHA-hyperforin	hyperforin dicyclohexylammonium salt
5-LO	5-lipoxygenase
COX	cyclooxygenase
PGE_2_	prostaglandin E2
fMLP	*N*-formyl-methionyl-leucyl-phenylalanine
LPS	lipopolysaccharide
PMN	polymorphonuclear neutrophils
PXR	pregnane X receptor
RXR	retinoid X receptor
HFD	high-fat-diet
AD	Alzheimer’s disease
SOD	superoxide dismutase
Aβ	amyloid-β-peptide
AAP	amyloid precursor protein
AST	aspartate aminotransferase
ALT	alanine aminotransferase
ICAM	intercellular cell adhesion molecule
BCL-2	B-cell lymphoma protein 2
BAX	B-cell lymphoma protein 2 (Bcl-2)-associated X
CXCL	C‑X‑C motif chemokine ligand
CIITA	immune-transcriptional co-activator
Puma	p53-upregulated mediator of apoptosis
Bim	B-cell lymphoma 2 interacting mediator of cell death
Chop	C/EBP homologous protein
PDX-1	pancreatic duodenal homeobox-1
FFA	free fatty acids
IAPP	islet amyloid polypeptide
FATP1	transporter of free fatty acids
HYP	hypericin
HFHS	high-fat/high-sucrose
NAFLD	non-alchoholic liver fatty disease
pAMPK	phosphorylated adenosine monophosphate-activated protein kinase
PKACs	protein kinase A catalytic
CRP	C reactive protein
TG	triglycerides
LDL	low-density lipoprotein
PKA	protein kinase cAMP-dependent
TGF-β	transforming growth factor beta
TRPC6	transient receptor potential channel
GABA	gamma-aminobutyric acid
NMDA	*N*-methyl-d-aspartate
PPAR-γ	peroxisome proliferator-activated receptor-gamma
STZ-NA	streptozotocin-nicotinamide
AGE	advanced glycation end products
TLR	Toll-like receptor
CYPs	cytochromes P450s
P-gp	P-glycoprotein
cAMP	cyclic adenosine monophosphate
PTP1B	protein tyrosine phosphatase 1B
